# Genome sequences of seven bacterial isolates collected from soil as part of a microbiology lab course

**DOI:** 10.1128/MRA.00357-23

**Published:** 2023-12-01

**Authors:** Belle Sharon, Tamineh Ebraimzadeh, Haley Dahl, Nymisha Avadhanam, Divya Rajendran, Ada Abaragu, Dylan Nguyen, Long Phan, Christine Ramjee, Mabel Thai, Gabrielle Vragel, Spring 2019 and Fall 2019 BIOL3520 Working Groups, Iti Mehta, Kelli Palmer

**Affiliations:** 1 Department of Biological Sciences, University of Texas at Dallas, Richardson, Texas, USA; Loyola University Chicago, Chicago, Illinois, USA

**Keywords:** soil microbiology, genomics, tiny earth

## Abstract

Soil is a source for diverse microbes that possess useful biotechnological capabilities. Here, we report the genome sequences of seven bacterial isolates from the species *Exiguobacterium acetylicum*, *Rossellomorea marisflavi*, *Delftia acidovorans*, *Pseudomonas aeruginosa*, *Bacillus* sp., and *Bacillus toyonensis* (two isolates) cultured from Dallas/Fort Worth metroplex soil samples.

## ANNOUNCEMENT

Soil is a useful source of diverse microbes that produce secondary metabolites of biotechnological utility (1, 2), and many clinically useful antibiotics originate from soil microbes (2, 3). The University of Texas at Dallas participates in the Tiny Earth curriculum, a course-based research experience that engages students in antibiotic discovery (3). Here, we report the genome sequences of seven bacterial isolates obtained by students from Dallas/Fort Worth area soils.

Soil samples were collected from the Dallas/Fort Worth metroplex area in Spring and Fall 2019 semesters by students. Surface soil was collected using sterile 50-mL conical tubes. One gram of soil was serially diluted using sterile deionized water. All bacterial isolates reported here were obtained by plating dilutions on 10% tryptic soy agar with 25 µg/mL cycloheximide, except for isolate UTDS19-33BHI26, which was obtained by plating on brain-heart infusion agar (BHI) with 25 µg/mL cycloheximide. Soil cultures were incubated at room temperature in ambient atmosphere for up to 1 week. Seven isolates were chosen for genome sequencing.

For DNA isolation, bacterial strains were inoculated in BHI broth and incubated at 37°C overnight. Genomic DNA was extracted using the DNeasy Blood and Tissue kit (Qiagen). DNA integrity was assessed using 1% agarose gel electrophoresis. DNA purity and yield were measured using Nanodrop 260/280 nm absorbance ratio and Qubit Fluorometer. The same extracted gDNA was used to perform both Illumina and Nanopore sequencing.

Illumina sequencing was performed by the Microbial Genome Sequencing Center using the Illumina Nextera kit for library preparation and NextSeq 550 platform for paired-end (2 × 150 bp) sequencing. Unless otherwise specified, default parameters were used for all software. Illumina reads were assessed and trimmed using CLC Genomics Workbench v12.0.3 to retain reads of Phred score ≥20 and read length >15 bp. Oxford Nanopore Technologies (ONT) MinION sequencing was used to generate long reads for hybrid genome assembly. Isolate libraries were prepared using the ligation sequencing kit (SQK-LSK109) and barcode expansion kit 1-12 (EXP-NBD104) and sequenced on R9 FLO-MIN106 ﬂow cells. ONT MinIT v19.12.5 [Guppy v3.2.10 (4)] was used for basecalling, demultiplexing, and barcode trimming. ONT read quality was assessed and filtered using NanoStats v1.2.0 (5) and NanoFilt v2.6.0 (5), respectively, to discard reads with a Phred score <7 and length <200 bp (6) (Table 1).

**TABLE 1:** Bacterial genomes sequenced in this study

	Strain	Taxonomic assignment	BioSample accession no.	SRA accession no. (O: ONT, I: Illumina)	Total no. of raw reads	Read depth (x)	Read N50 (bp)	GenBank/WGS accession no.	No. of contigs	Type of contig	Total length (bp)	GC content (%)	CDS no.

Complete genome assembly	UTDF19-27C	*Exiguobacterium acetylicum*	SAMN20890001	I: SRX13285893	8,809,238	317.7		CP081878	2	Chromosome	3,176,680	46.80%	3,223
				O: SRX13289959	41,775	45	5,958	CP081879		Contig 1	184,468	45.90%	164
	UTDF19-29B	*Bacillus toyonensis*	SAMN20890002	I: SRX13285894	11,736,568	235.1		CP081872	6	Chromosome	5,240,743	35.40%	5,175
				O: SRX13289960	66,199	35.6	5,744	CP081873		Contig 1	350,788	32.40%	319
								CP081874		Contig 2	262,462	32.80%	235
								CP081875		Contig 3	13,279	30.50%	14
								CP081876		Contig 4	3,188	32.90%	3
								CP081877		Contig 5	1,764	37.80%	2
	UTDF19-31A	*Rossellomorea marisflavi*	SAMN20890003	I: SRX13285895	8,872,678	232.1		CP081870	2	Chromosome	4,299,345	48.30%	4,316
				O: SRX13289961	157,208	65.2	4,916	CP081871		Contig 1	10,968	39.30%	9
Draft genome assembly	UTDF19-28A	*Pseudomonas aeruginosa*	SAMN23567015	I: SRX13285896	6,911,580	119.9		JAJPGI000000000	6	Contig 1	2,961,111	66.60%	2,709
				O: SRX13289962	115,747	50.4	5,372			Contig 2	2,328,118	66.00%	2,125
										Contig 3	632,753	65.90%	591
										Contig 4	347,323	67.40%	247
										Contig 5	135,866	65.30%	133
										Contig 6	37,975	65.90%	33
	UTDS19-33BHI26	*Bacillus* sp.	SAMN23567016	I: SRX13285897	8,968,014	193.6		JAJPGH000000000	9	Contig 1	5,264,481	35.40%	5,316
				O: SRX13289963	800,838	195.3	2,135			Contig 2	303,603	33.80%	298
										Contig 3	74,405	35.10%	61
										Contig 4	14,055	32.90%	9
										Contig 5	6,956	33.80%	6
										Contig 6	3,783	34.50%	3
										Contig 7	3,166	33.10%	3
										Contig 8	3,052	35.40%	3
										Contig 9	1,282	36.70%	2
										Contig 10	130	23.80%	0
	UTDS19-34TSA4	*Bacillus toyonensis*	SAMN23567017	I: SRX13285898	12,416,682	251.5		JAJPGG000000000	7	Contig 1	5,222,960	35.40%	5,189
				O: SRX13289964	1,078,479	205.8	1,797			Contig 2	297,388	32.60%	276
										Contig 3	282,870	32.60%	248
										Contig 4	56,560	30.60%	57
										Contig 5	52,523	34.20%	67
										Contig 6	18,930	34.40%	18
										Contig 7	1,100	34.80%	2
										Contig 8	152	32.20%	0
	UTDS19-35T5	*Delftia acidovorans*	SAMN23567018	I: SRX13285899	8,693,252	150.2		JAJPGF000000000	5	Contig 1	3,356,667	66.90%	2,960
				O: SRX13289965	41,276	20.6	8,192			Contig 2	1,679,814	66.50%	1,482
										Contig 3	1,209,792	67.60%	1,109
										Contig 4	226,467	65.40%	191
										Contig 5	30,072	68.10%	24

For each strain, Illumina and ONT reads were hybrid assembled using Unicycler v0.4.8 (SPAdes v3.13.0, Racon v1.4.10, and Pilon v1.23) at default parameters (7 – 11). Hybrid assembly quality was evaluated using QUAST v5.0.2 (12), and genome completeness was assessed using Bandage v0.8.1 (13) and BUSCO v1 (14) with bacteria ortholog set on gVolante server v1.2.1 (15). Three genome assemblies were complete, whereas four assemblies remain draft. Circular genomic sequences were rotated in Unicycler to the starting base of *dnaA* or *repA*, if found. Hybrid assemblies were annotated using the NCBI Prokaryotic Genome Annotation Pipeline v4.11 (16). GC content and coding sequences number were obtained using Geneious Prime v2022.1.1 (Table 1).

## Data Availability

Sequences were submitted to GenBank as part of BioProject PRJNA756567. Further details and accession numbers are described in Table 1.
